# Recombinant *Escherichia coli*-driven whole-cell bioconversion for selective 5-Aminopentanol production as a novel bioplastic monomer

**DOI:** 10.1186/s40643-025-00904-6

**Published:** 2025-06-10

**Authors:** Byung Wook Lee, Hee Taek Kim, Hyun Gi Koh, Kyungjae Yu, Gaeul Kim, Yoon Jung Jung, Haeng-Geun Cha, Yunhee Jeong, Yung-Hun Yang, See-Hyoung Park, Kyungmoon Park

**Affiliations:** 1 Department of Biological and Chemical Engineering, Hongik University, Sejong, 30016 Republic of Korea; 2 Department of Food Science and Technology, Chungnam National University, Daejeon, 34134 Republic of Korea; 3 Department of Biological Engineering, Konkuk University, Seoul, 05029 Republic of Korea

**Keywords:** 5-Aminopentanol, l-Lysine (C6) valorization process, Whole-cell bioconversion, *Escherichia coli*, Novel bioplastic monomer

## Abstract

5-Aminopentanol (5-AP) is a valuable amino alcohol with potential applications in polymer synthesis and bioplastics. Conventional production methods rely on petroleum-based feedstocks and metal catalysts, which raise environmental and sustainability concerns. In this study, a de novo biosynthetic pathway for 5-AP production from l-lysine was developed in *Escherichia coli*. The engineered pathway consisted of lysine decarboxylase 2 (LdcC), putrescine aminotransferase (PatA), and tested aldehyde reductase (YahK, YihU, YqhD). Among the tested reductases, aldehyde reductase exhibited the highest catalytic efficiency, producing 44.5 ± 2.6 mM of 5-AP (0.44 ± 0.03 mol_5 − AP_/mol_l−lysine_). The replacement of the expression system with a T7-based dual-plasmid platform, pET24ma::*ldcC*, and pCDFDuet-1::*yqhD*::*patA* co-transformed into *E. coli*, increased the production to 60.7 ± 5.8 mM, accompanied by reduced cadaverine accumulation. Further enhancement was achieved by increasing the gene dosage of PatA, leading to 68.5 ± 4.2 mM 5-AP and reduced by 40% in cadaverine levels. Cadaverine is a precursor in the production of 5-AP, and its accumulation is an important factor in the limitation of conversion to 5-AP. Intracellular cofactor regeneration is expected to cause an indirect supply of α-KG, a cofactor, to enhance conversion to 5-AP. To support intracellular cofactor regeneration, glucose supplementation and increased aeration were applied, resulting in a final titer of 78.5 ± 1.2 mM 5-AP and improved precursor utilization. This study is the first report of selective microbial 5-AP production and highlights the importance of PatA expression in pathway optimization. The newly established l-lysine (C6) valorization process which converts l-lysine to high-value materials such as 1,5-PDO, glutarate, and 5-AP offers a promising route for the sustainable biosynthesis of amino alcohols, laying the groundwork for future improvements through enzyme engineering and metabolic design.

**Supplementary Information:**

The online version contains supplementary material available at 10.1186/s40643-025-00904-6.

## Introduction

The increasing environmental pollution caused by the excessive production and use of petroleum-based plastics has led to a growing interest in the development of bioplastics (Millican and Agarwal 2021; MacLeod et al. 2021). Non-biodegradable plastics, which cannot be degraded into CO_2_ through natural remediation processes, have accumulated over time, leading to various plastic waste problems. Furthermore, microplastics, generated through physicochemical degradation without further breakdown into CO_2_, have caused serious detrimental impacts on ecosystems, human health, and climate change (Akdogan and Guven 2019; Amobonye et al. 2021; Zhang et al. 2022). These issues have highlighted the urgent need for the development of more environmentally friendly plastics, such as bio-based polymers. Accordingly, various plastics, including polyesters and polyamides, have been successfully developed using cadaverine and glutaric acid produced by recombinant microorganisms. These bioplastics are synthesized from renewable resources, such as glucose and l-lysine. Recently, as part of these research efforts, a new type of biodegradable plastic, polyurea ester (PUE), has been proposed (Tang et al. 2016; Dziewior et al. 2024).

PUE is emerging as a promising candidate to address the poor mechanical properties that have limited conventional biodegradable polymers. Its production begins with the synthesis of di(hydroalkyl)urea via the reaction of amino alcohols, such as 4-aminobutanol, 5-aminopentanol (5-AP), and 6-aminohexanol with urea, followed by polymerization with dimethyl esters (e.g., dimethyl succinate, adipate, or sebacate) to form PUE. The production of PUE requires amino alcohols like 5-AP as key precursors for prepolymer synthesis. Given these advantages, there is growing interest in the biological production of amino alcohols such as 5-AP. This approach offers a promising route toward sustainable and environmentally friendly PUE production by reducing reliance on petroleum-based processes and utilizing bio-based substrates and biological methods (López-Garzón et al. 2014; Prabowo et al. 2020; Wang et al. 2023).

5-AP is a linear C5 chemical with an amino group at one end and a hydroxyl group at the other. Traditionally, its production has relied on chemical methods, using furfural as substrate and Ni-based catalysts, such as NiCo/Al₂O₃ and Ni-Mg₃AlOx_2_, to facilitate the reaction (Li et al. 2020; Yang et al. 2024). Although this method offers high conversion efficiency, the use of metal-based catalysts raises environmental and human health concerns, emphasizing the need for a more sustainable production route (Egorova and Ananikov 2016; Das et al. 2019). Conventionally, 5-AP is utilized in the chemical synthesis of δ-valerolactam, a monomer or precursor for various polymers such as nylon-5 (Reddy et al. 1994; Nova et al. 2010; Von Tiedemann et al. 2020). More recently, 5-AP has gained attention as a monomer for di(hydroxyalkyl)urea, which can be polymerized with diacids, such as dimethyl succinate, dimethyl adipate, and dimethyl sebacate, to produce a novel polymer, PUE. Despite its expanding applications, there have been no reports on the selective biosynthesis of 5-AP with high conversion efficiency from renewable resources such as glucose and l-lysine.

An l-lysine (C6) valorization process which converts l-lysine to high-value materials such as 1,5-PDO, glutarate, and 5-AP was successfully developed for producing C5 building blocks using a whole-cell bioconversion approach. This includes the biological production of compounds such as cadaverine, glutaric acid, and 1,5-pentanediol. The valorization of cadaverine, a decarboxylated diamine derivative of l-lysine, has been reported to reach titers exceeding 2 M in *Escherichia coli* strains expressing l-lysine decarboxylases, such as lysine decarboxylase 1 (CadA) and lysine decarboxylase 2 (LdcC) (Oh et al. 2015; Kim et al. 2019). Glutaric acid production, another successfully developed l-lysine valorization process, can be achieved through a sequential enzymatic reaction involving lysine monooxygenase, δ-aminovaleramidase, 5-aminovalerate aminotransferase, and glutarate semialdehyde dehydrogenase. Initial studies achieved a glutaric acid production yield of 1.7 g/L through the whole-cell bioconversion of l-lysine using an *E. coli* strain (Rohles et al. 2016). More recently, the yield was substantially improved, with over 70 g/L of glutaric acid produced using *E. coli* (Wang et al. 2021). 1,5-Pentanediol (1,5-PDO) has also been reported to be produced via whole-cell bioconversion by implementing a novel biosynthetic route from l-lysine to 1,5-PDO in an engineered *E. coli* strain, achieving a concentration of 4.03 mM. To enable this biosynthetic route, CadA, 4-aminobutyrate aminotransferase, NADPH-dependent reductase, and glutamate dehydrogenase were introduced into *E. coli*. This engineering strategy enabled the successful synthesis of 1,5-PDO through the sequential conversion of l-lysine via decarboxylation, cadaverine transamination, and glutaraldehyde reduction, with cofactor regeneration further supporting and enhancing 1,5-PDO production (Hua et al. 2024).

Despite the successful biosynthesis of various C5 chemicals, including cadaverine (as a diamine), glutaric acid (as a diacid), and 1,5-pentanediol (as a dialcohol), the selective biological production of 5-AP as an amino alcohol has not yet been reported. In this study, a selective and efficient biosynthetic pathway for 5-AP production from l-lysine was established, representing the first reported microbial route with a high conversion yield. A de novo biosynthetic pathway was constructed to enable the stepwise conversion of l-lysine to 5-AP. Among several candidates, a reductase responsible for converting 5-aminopentanal to 5-aminopentanol was identified and incorporated into the system. To improve production efficiency, key reaction parameters including temperature, pH, and concentrations of cofactors and co-substrates were systematically evaluated. Based on these analyses, the transamination step catalyzed by PatA was identified as a major bottleneck. To address this, the gene copy number of the *patA* was increased and α-ketoglutarate (α-KG) was supplemented, leading to enhanced flux toward 5-AP via more efficient cadaverine conversion.

## Materials and methods

### Chemicals

Chemicals used in this study, including l-lysine (≥ 98%), cadaverine (≥ 95%), 5-aminopentanol (≥ 92%), α-KG (≥ 99%), isopropyl β-D-1-thiogalactopyranoside (IPTG; ≥99%), pyridoxal-5-phosphate (≥ 99%), NADPH, tetrasodium salt (≥ 93%), imidazole (≥ 99%), potassium phosphate dibasic (≥ 98%), kanamycin, and ampicillin, were purchased from Sigma-Aldrich (St. Louis, MO, USA). Tris-HCl (pH 8.5) was purchased from LPS Solutions (Daejeon, Korea). High salt lysogenic broth and spectinomycin were purchased from Duchefa Biochemie (Haarlem, Netherlands) and Tokyo Chemical Industry (Tokyo, Japan), respectively. Diethyl ethoxymethylenemalonate (97%) and methanol (≥ 99.9%) were purchased from Tokyo Chemical Industry and Sigma-Aldrich. Water, sulfuric acid (≥ 99.999%), 1 M sodium acetate (pH 4.6), and acetonitrile were purchased from Samchun Chemicals (Seoul, Republic of Korea), Sigma-Aldrich, Biosolution (Seoul, Republic of Korea), and Avantor (Pennsylvania, USA), respectively, and were used in the preparation of the high-performance liquid chromatography (HPLC) mobile phase.

### Plasmids and bacterial strains

*E. coli* DH5α was used for genetic manipulation, while BL21(DE3) was employed for whole-cell catalyst preparation. DNA manipulation was performed according to the standard protocols. For the initial biosynthetic route construction of 5-AP biosynthesis, the customized vector pKM212 was used to generate pKM212::*ldcC*::*patA*::*yahK*, pKM212::*ldcC*::*patA*::*yihU*, and pKM212::*ldcC*::*patA*::*yqhD*. After digestion with the restriction enzymes specified in the primer list, the constructs were assembled using the primers listed in Supplementary Table 2. Additionally, the derivative vector pET24ma, which was modified from pET24a by replacing f1 *ori* with p15A *ori*, was used to construct pET24ma::*ldcC* by ligating the amplified *ldcC* gene. The gene products were amplified using the primers listed in Supplementary Tables 2 and subsequently digested with the restriction enzymes listed in Supplementary Table 2 (Shin et al. 2018). Gibson Assembly® Master Mix from New England Biolabs (E2611L) was used for assembling pCDFDuet-1::*yqhD*::*patA*, and pET21b(+)::*patA*. Commercial vectors and genes, pCDFDuet-1, pET21b(+), *yqhD*, and *patA* were amplified (Gibson et al. 2009). The information about all genes used in this study is listed in Supplementary Table (1) The amplified vectors and genes were assembled according to the manufacturer’s instructions. The primers used to amplify the vectors and genes are listed in Supplementary Table (2) The constructed vectors are listed in Supplementary Table (3) The plasmid maps are provided in Supplementary Fig. 1.

**Fig. 1:**
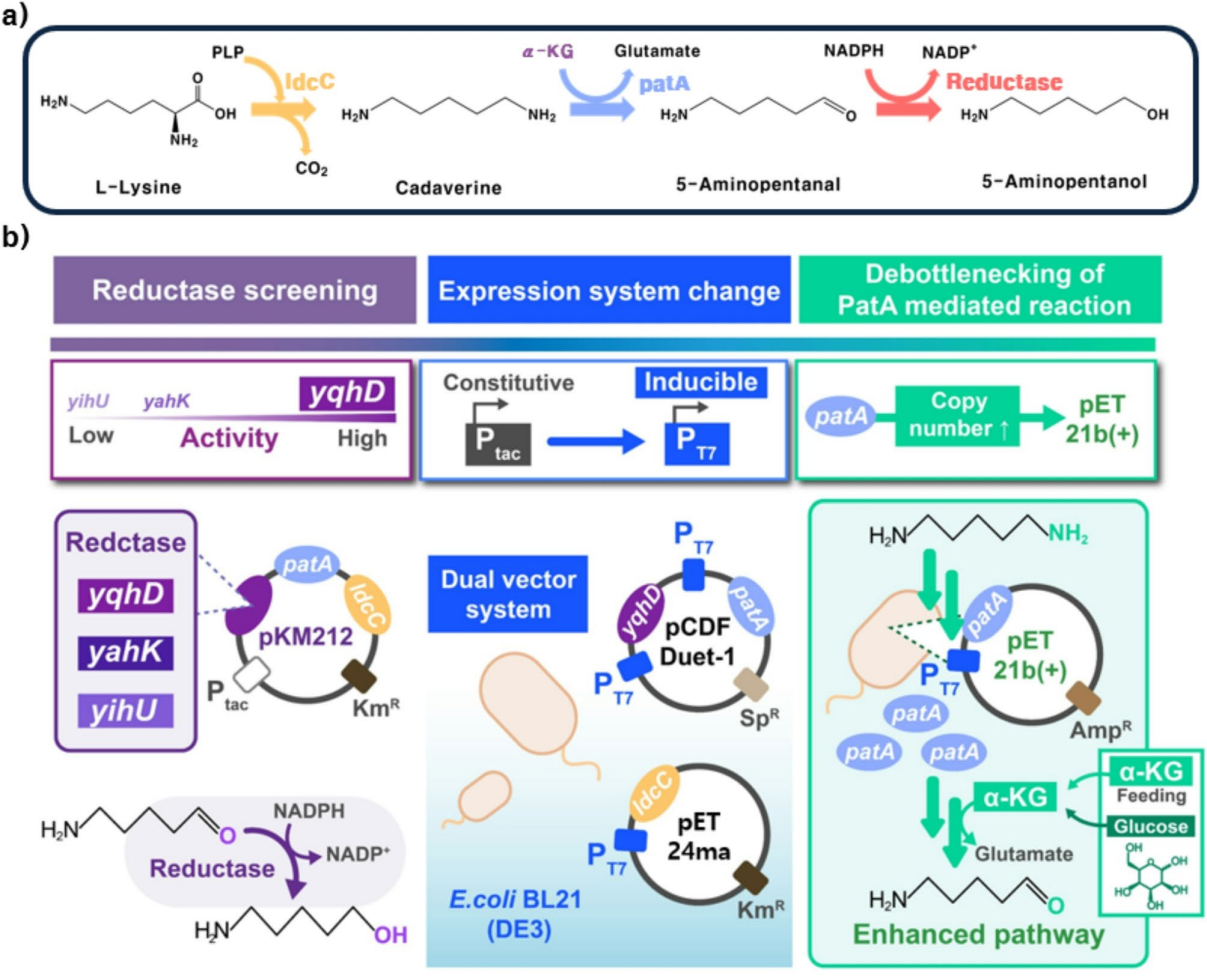
Strategies for developing a biosynthetic system for 5-AP production from l-lysine. **(a)** Schematic representation of the whole-cell bioconversion pathway from l-lysine to 5-AP. **(b)** Overview of the engineering strategies employed in this study for constructing the 5-AP biosynthesis platform

### Whole-cell biocatalyst preparation and reaction

For the heterologous expression of enzymes to prepare biocatalysts, seed culture was performed using the recombinant *E. coli* strains listed in Supplementary Table 3 at 37 °C and 200 rpm overnight. The seed culture (4 mL) was transferred to 100 mL of culture medium and incubated at 30 °C with shaking at 200 rpm for 18–24 h. When the optical density (OD_600_) reached 0.6, 0.05 mM isopropyl β-D-1-thiogalactopyranoside was added to the culture medium, and the cells were further cultivated at 16 °C with shaking at 200 rpm for 24 h. The biocatalysts were harvested by centrifugation at 4 °C and 4000 rpm for 20 min, followed by two washes with 1 M Tris-HCl buffer (pH 8.5). For the preparation of the whole-cell bioconversion reaction, reaction mixtures were prepared in 5 mL of 500 mM Tris-HCl buffer (pH 8.5), containing 100 mM l-lysine, α-KG (20–100 mM), NADPH (0 or 10 mM), and 0.1 mM pyridoxal-5-phosphate. When necessary, glucose (0–4%), mannitol (2%), and sorbitol (2%) were added to the reaction mixtures. The standard whole-cell bioconversion reactions were carried out in 50-mL falcon tubes at pH 6.5 and 30 °C for 48 h (OD_600_ = 40). Sampling was conducted every 24 h. After each sampling, the samples were centrifuged to obtain a culture medium containing l-lysine, cadaverine, and 5-AP.

### Analytic method

Cell density was measured by determining OD_600_ using a spectrophotometer (X-ma 1200 V; Human Corporation, Seoul, Republic of Korea). The concentration of α-KG was quantified using HPLC (Nexera, Shimadzu, Tokyo, Japan) equipped with a refractive index detector and an Aminex HPX-87 H column (300 × 7.8 mm, Bio-Rad, California, USA), using a flow rate of 0.6 mL/min at 60 °C. Sulfuric acid (4 mM) was used as the mobile phase. The concentrations of l-lysine, cadaverine, and 5-AP were determined using HPLC with a Capcell Pak C18 UG (Agilent ZORBA × SD-C18 column, California, USA) and the diethyl ethoxymethylenemalonate derivatization method. Potassium borate buffer, methanol, distilled water, diethyl ethoxymethylenemalonate, and sample were mixed together. For 2 h, the samples were heated at 70 °C (Kim et al. 2015). Peaks for each chemical were identified at a wavelength of 284 nm using a variable wavelength detector. Peaks were separated through gradient elution with mobile phases consisting of 25 mM sodium acetate buffer (pH 4.8) as buffer A and 100% acetonitrile as buffer B, using the following gradient program: 0–2 min, 20–25% buffer B; 2–32 min, 25–60% buffer B; and 30–40 min, 60–20% buffer B. The flow rate was set to 1 mL/min, and the column oven temperature was maintained at 40 °C (Cha et al. 2022, 2023). Conversion yield and selectivity were calculated using the following equations, respectively.

$$Conversion\>yield\left( \% \right) = {\matrix{ Concentration\>of\>5 - \hfill \cr AP\>after\>convesion\>\left( {mM} \right) \hfill \cr} \over \matrix{ Concentration\>of\>l - \hfill \cr lysine\>before\>convesion\>\left( {mM} \right) \hfill \cr} }$$

$$\eqalign{& Conversion\>selectivity\left( \% \right) = \cr & {\matrix{ Concentration\>of\>5 - \hfill \cr AP\>after\>convesion\>\left( {mM} \right) \hfill \cr} \over \matrix{ Concentration\>of\>l - \hfill \cr lysine\>after\>convesion\>\left( {mM} \right) + \hfill \cr Concentration\>of\>cadaverine\>after\>convesion \hfill \cr} }\cr}$$

## Results and discussion

### Development of 5-AP biosynthetic pathway from l-lysine

**Fig. 2:**
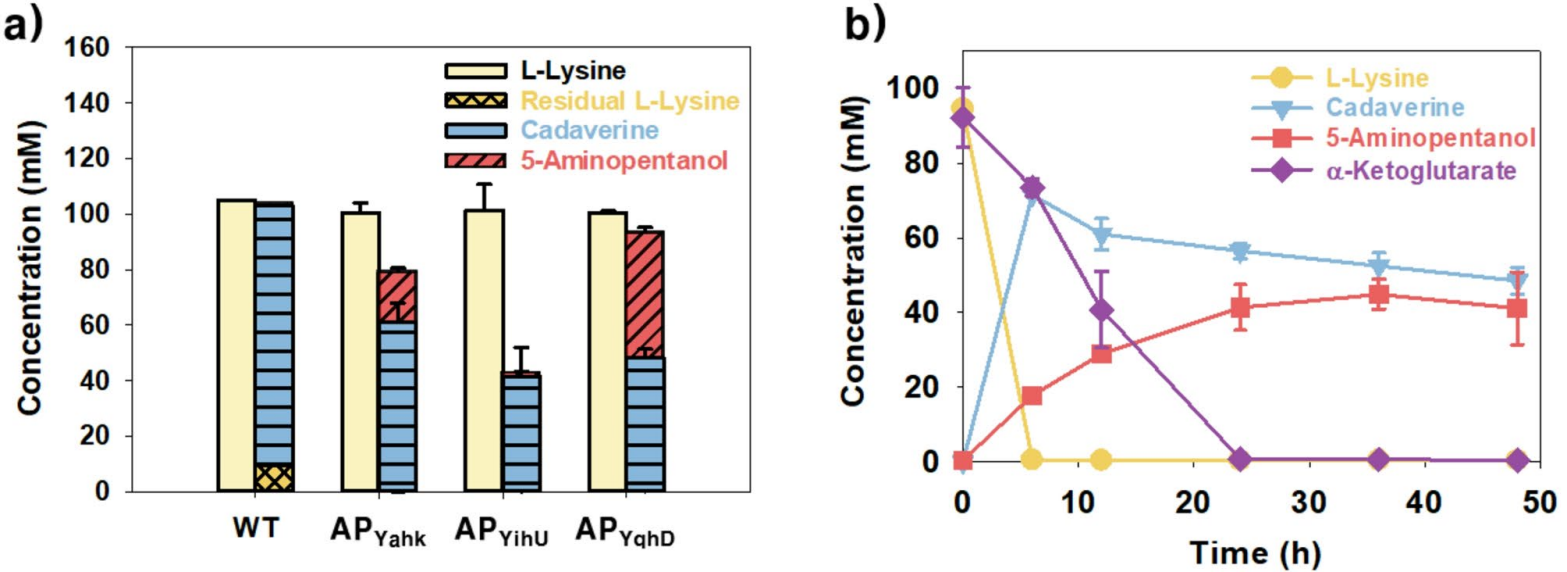
Screening of efficient reductases for the reduction of 5-aminopentanal. **(a)** Comparison of 5-AP production from l-lysine using *E. coli* AP_YahK_, *E. coli* AP_YihU_, and *E. coli* AP_YqhD_, expressing different reductases at 36 h. Beige bars: concentration of l-lysine at 0 h, yellow diamond bars: concentration of residual l-lysine at 36 h, blue ladder bars: concentration of cadaverine at 36 h, orange diagonal bars: concentration of 5-AP at 36 h **(b)** Time-course profile of 5-AP production from l-lysine in *E. coli* AP_YqhD_

5-AP is typically produced through chemical conversion processes using both petroleum-based and biomass-derived substrates, such as olefins and furfural. Although these methods have demonstrated efficient production, they pose significant environmental concerns owing to the use of metal catalysts like NiCo/Al₂O₃ and Ni-Mg₃AlOx_2_ (Pelckmans et al. 2017; Li et al. 2020; Yang et al. 2024). Recently, environmentally friendly biological methods for 5-AP synthesis from glucose have been developed using *E. coli* expressing CadA, PatA, and alcohol dehydrogenase (YjgB). However, because the system was originally designed for 1,5-pentanediol production, 5-AP was only produced as an intermediate, resulting in a low titer (1.5 g/L) and poor selectivity (Ma et al. 2025). To establish an environmentally friendly and more selective biological platform for 5-AP production, a biosynthetic pathway starting from l-lysine was developed in this study. This design was guided by the mechanistic similarity between the l-lysine-to-5-AP route and the established conversion of putrescine to 4-aminobutanol, which led to the incorporation of cadaverine as a key intermediate (Figure. 1a). As shown in Fig. 1, *ldcC* and *patA*, which encode lysine decarboxylase 2 and diamine aminotransferase, respectively, were introduced into the host strain. To screen for the most efficient reductase for the conversion of 5-aminopentanal to 5-AP, three candidate enzymes (*yahK*, *yihU*, and *yqhD*) were individually introduced into the pKM212::*patA*::*ldcC* backbone, resulting in the constructs pKM212::*yahK*::*patA*::*ldcC*, pKM212::*yihU*::*patA*::*ldcC*, and pKM212::*yqhD*::*patA*::*ldcC* (Figure. 1b). Each plasmid was then transformed into *E. coli* BL21(DE3) cells for gene overexpression, resulting in *E. coli* AP_YahK_, *E. coli* AP_YihU_, and *E. coli* AP_YqhD_. Whole-cell bioconversion was performed using the recombinant strains *E. coli* AP_YahK_, *E. coli* AP_YihU_, and *E. coli* AP_YqhD_ with 100 mM l-lysine and 100 mM α-KG in 500 mM Tris-HCl buffer (pH 6.5) at 30 °C, resulting in 5-AP production of 15.6 ± 2 mM, 2.2 ± 0.2 mM, and 44.5 ± 0.03 mM, respectively (Fig. 2a). The conversion profile of YqhD revealed that l-lysine was completely consumed within 6 h, with the concurrent formation of cadaverine (71.4 ± 0.4 mM) and 5-AP (17.7 ± 1.2 mM), followed by the gradual depletion of cadaverine and a peak in 5-AP concentration at 36 h (44.5 ± 2.6 mM), after which a slight decrease was observed (Fig. 2b). Accordingly, YqhD was identified as the most effective enzyme in the biosynthetic pathway with the highest 5-AP conversion yield (0.44 ± 0.03 mol_5 − AP_/mol_l−lysine_). This observation aligns with previous reports demonstrating the superior catalytic performance of YqhD over other reductases in the production of C4 and C5 compounds, such as γ-hydroxybutyrate and 1,5-pentanediol. In the case of γ-hydroxybutyrate production, YqhD was reported to be more than twice as effective as other reductases, including YjgB, YahK, and YihU (Zhu et al. 2024). Similarly, for 1,5-pentanediol production, YqhD exhibited approximately 1.5-fold higher activity than other reductases such as YjgB and YahK (Jarboe 2011; Cen et al. 2022). Taken together, these results demonstrate that the newly constructed biosynthetic pathway from l-lysine to 5-AP was functionally active.

### Enhanced 5-AP production through adjustment of the expression system

**Fig. 3:**
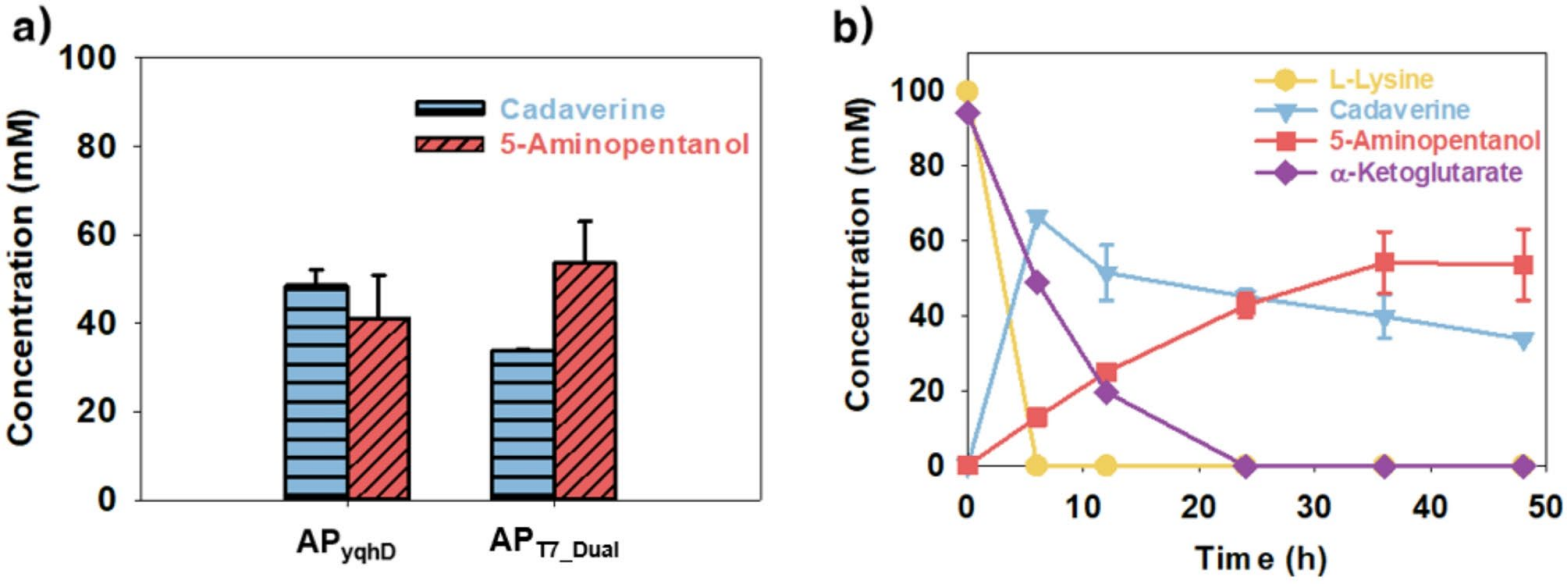
Enhancing conversion efficiency through modification of the expression system. **(a)** Effect of expression system modification on 5-AP production from l-lysine using *E. coli* AP_T7_Dual_ at 36 h. **(b)** Time-course profile of 5-AP production and cadaverine accumulation in *E. coli* AP_T7_Dual_

Despite the successful conversion of l-lysine to 5-AP using *E. coli* AP_YqhD_, the yield was limited to 0.44 ± 0.03 mol_5 − AP_/mol_l−lysine_. To address this limitation, the Tac promoter–based custom vector pKM212 was replaced with T7 promoter–driven expression vectors (pCDFDuet-1 and pET24ma), as the T7 system is widely used for the high-level expression of recombinant proteins supported by bacteriophage T7 RNA polymerase (Lozano Terol et al. 2021). Using this system, a dual-enzyme expression vector (pCDFDuet-1::*yqhD*::*patA*) and a single-enzyme expression vector (pET24ma::*ldcC*) were constructed and co-transformed into *E. coli* BL21(DE3), resulting in *E. coli* AP_T7_Dual_ harboring both plasmids (Fig. 1b). To compare 5-AP production efficiency, whole-cell bioconversion was conducted using *E. coli* AP_YqhD_ and *E. coli* AP_T7_Dual_ under the same reaction conditions (100 mM l-lysine and 100 mM α-KG in 500 mM Tris-HCl buffer at pH 6.5 and 30 °C). As a result, *E. coli* AP_YqhD_ and AP_T7_Dual_ produced 44.5 ± 2.6 and 60.7 ± 5.8 mM 5-AP, respectively (Fig. 3a). Although both strains completely consumed l-lysine within 6 h and showed similar initial trends, *E. coli* AP_T7_Dual_ continued to convert cadaverine even after 24 h, leading to a final 5-AP concentration of 60.7 ± 5.8 mM at 36 h, higher than that observed for *E. coli* AP_YqhD_ (Fig. 3b). Additionally, cadaverine accumulation was reduced in the *E. coli* AP_T7_Dual_ strain, with 40 ± 0.9 mM cadaverine accumulating, compared with 55 ± 2.5 mM in the *E. coli* AP_YqhD_ strain. These findings indicate that the change in the expression system contributed to enhanced 5-AP production, with *E. coli* AP_T7_Dual_ achieving a 1.36-fold improvement over *E. coli* AP_YqhD_ under the tested conditions. However, cadaverine accumulated partially unconverted at 48 h, indicating that the overall pathway had not yet reached full efficiency and may benefit from further optimization of the reaction conditions and pathway components (Fig. 3b).

### Evaluation of reaction conditions and pathway limitations in 5-AP biosynthesis

**Fig. 4:**
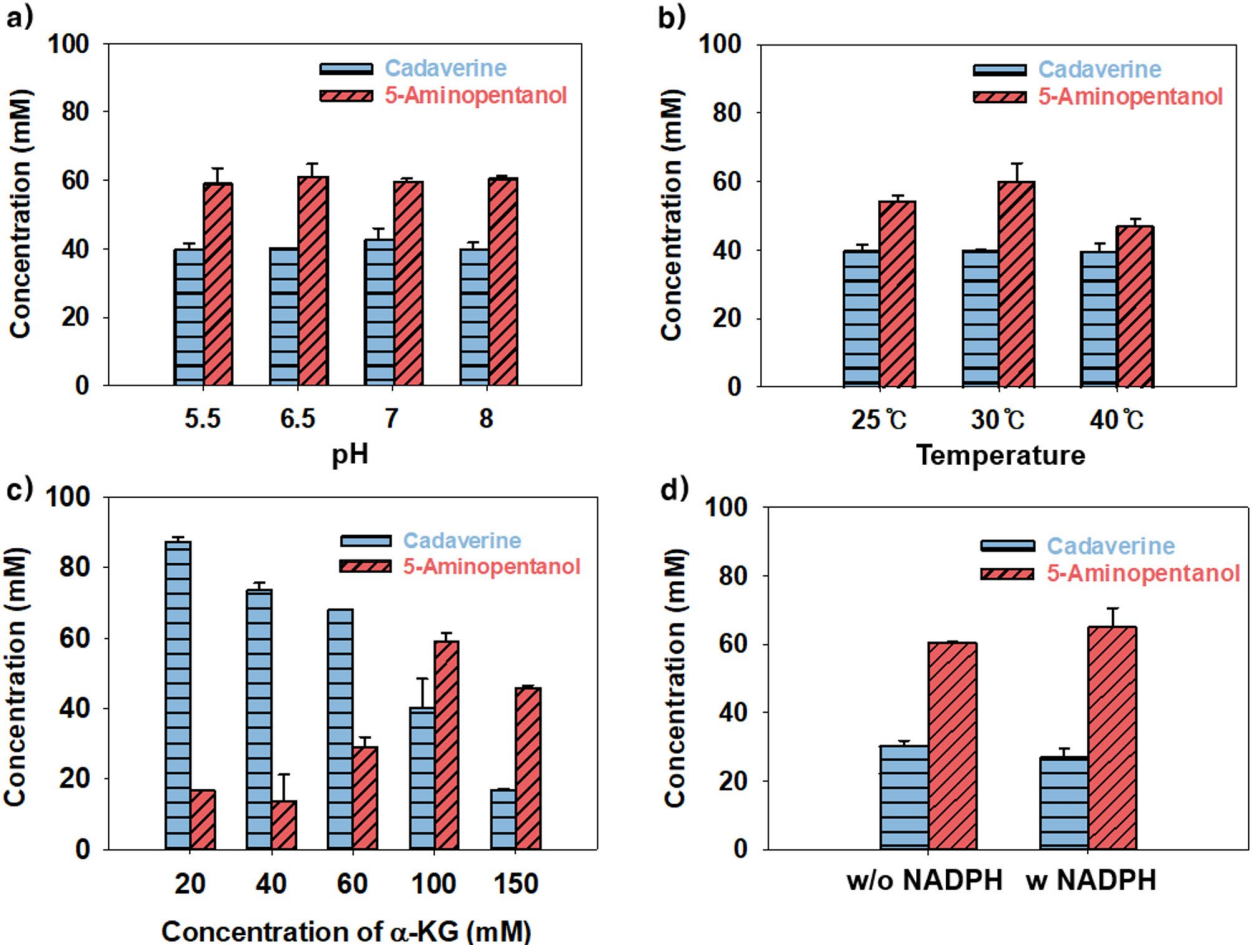
Identification of bottlenecks in whole-cell bioconversion. Effect of reaction conditions and cofactors on 5-AP production including **(a)** temperature, **(b)** pH, **(c)** concentration of α-KG, and **(d)** supplementation of NADPH. w/o NADPH shows the same result as the previous condition as a control, and w NADPH indicates the results when 10 mM of NADPH was supplied

To investigate the cause of residual cadaverine observed in the previous experiments, whole-cell bioconversion was carried out using *E. coli* AP_T7_Dual_ under varying reaction conditions, including pH, temperature, α-KG concentration, and NADPH supplementation (Fig. 4). Within the tested pH range (5.5 to 8.0), 5-AP production showed no significant variation, and temperature had only a modest effect, with 30 °C giving slightly higher titers than other conditions (Fig. 4a and b). In the case of α-KG concentration, 100 mM was found to be optimal, while higher concentrations led to a decrease in 5-AP production (Fig. 4c). Supplementation with 10 mM NADPH did not result in a significant increase in 5-AP production, indicating that cofactor addition alone was insufficient to improve the overall pathway efficiency (Fig. 4d). These optimal values coincided with the standard conditions used in this study, suggesting that the earlier bioconversion assays had already been conducted under near-optimal conditions. Figure 3b indicates that, although the reaction was conducted under favorable conditions, a substantial amount of cadaverine accumulated at the end of the process. Because cadaverine is the direct precursor of 5-AP, this observation suggests that the downstream steps, particularly the conversion of cadaverine to 5-aminopentanal and subsequently to 5-AP, were limiting under the tested conditions. These reactions are catalyzed by PatA and YqhD, which utilize α-KG and NADPH, respectively. Given that both cofactors were sufficiently supplied, this limitation was presumed to arise from inadequate enzymatic activity rather than substrate availability. Among the two enzymes, these PatA-mediated transamination step was considered the primary bottleneck, because it governs the flux from cadaverine to 5-aminopentanal, a key intermediate in the pathway. To address this limitation, the next set of experiments evaluated whether enhanced PatA expression could reduce cadaverine accumulation, thereby improving 5-AP production.

### Effect of PatA expression on the 5-AP production

**Fig. 5:**
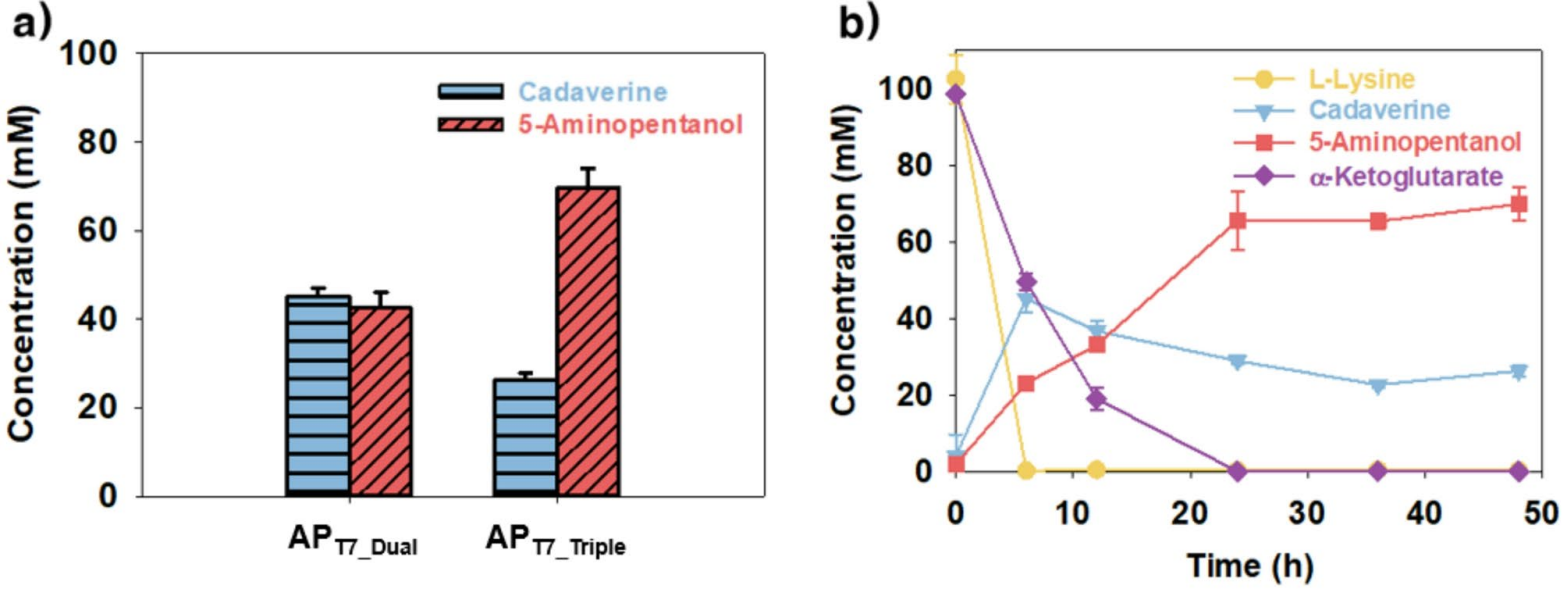
Effect of increasing the *patA* gene copy number. **(a)** Comparison of 5-AP production and cadaverine accumulation by *E. coli* AP_T7_Dual_ and *E. coli* AP_T7_Triple_ at 24 h. **(b)** Time-course profile of 5-AP production showing improved conversion by *E. coli* AP_T7_Triple_

Because adjustments to the reaction conditions did not significantly improve 5-AP production, the PatA-mediated transamination step was suspected to be a bottleneck, likely due to insufficient enzyme expression, which limited the conversion of cadaverine to 5-aminopentanal in the l-lysine-to-5-AP pathway. This hypothesis is supported by a previous study that reported that 4-aminobutanol production was improved by repositioning the *patA* gene within the plasmid and adding an N-terminal 6×His tag to increase expression levels (Prabowo et al. 2020). To address this issue, the effect of increasing the *patA* gene copy number was investigated (Fig. 5). A separate plasmid, pET21b(+)::*patA*, was constructed and co-transformed into *E. coli* AP_T7_Dual_, resulting in a new strain, *E. coli* AP_T7_Triple_ (Fig. 1b). Whole-cell bioconversion using this strain yielded 68.5 ± 4.2 mM of 5-AP from 100 mM l-lysine and 100 mM α-KG in 500 mM Tris-HCl buffer at 30 °C and pH 6.5, corresponding to a 68.5 ± 4.2% yield at 24 h. This was 1.6-fold higher than that produced by *E. coli* AP_T7_Dual_ at 24 h (42.8 ± 2.8 mM) (Fig. 5a, b). Cadaverine accumulation was also significantly reduced, with the residual concentration decreasing to 28.5 ± 0.9 mM, which was 0.63-fold of the level observed in *E. coli* AP_T7_Dual_ at 24 h (44.7 ± 1.3 mM) (Fig. 5a, b). In terms of molar conversion, the yield improved from 6.07 ± 0.58 mol_5 − AP_/mol_l−lysine_ in *E. coli* AP_T7_Dual_ to 6.85 ± 0.42 mol_5 − AP_/mol_l−lysine_ in *E. coli* AP_T7_Triple_. These results suggest that increased PatA expression, achieved by co-expressing *patA* from an additional plasmid, improved the efficiency of cadaverine conversion and utilization of α-KG. Collectively, these findings confirmed the importance of the PatA-catalyzed step as a key control point in the overall biosynthetic pathway. The resulting strain, *E. coli* AP_T7_Triple_, represents a significantly improved host for 5-AP production compared with earlier constructs.

### Enhancing 5-AP production via carbon source supplementation

**Fig. 6:**
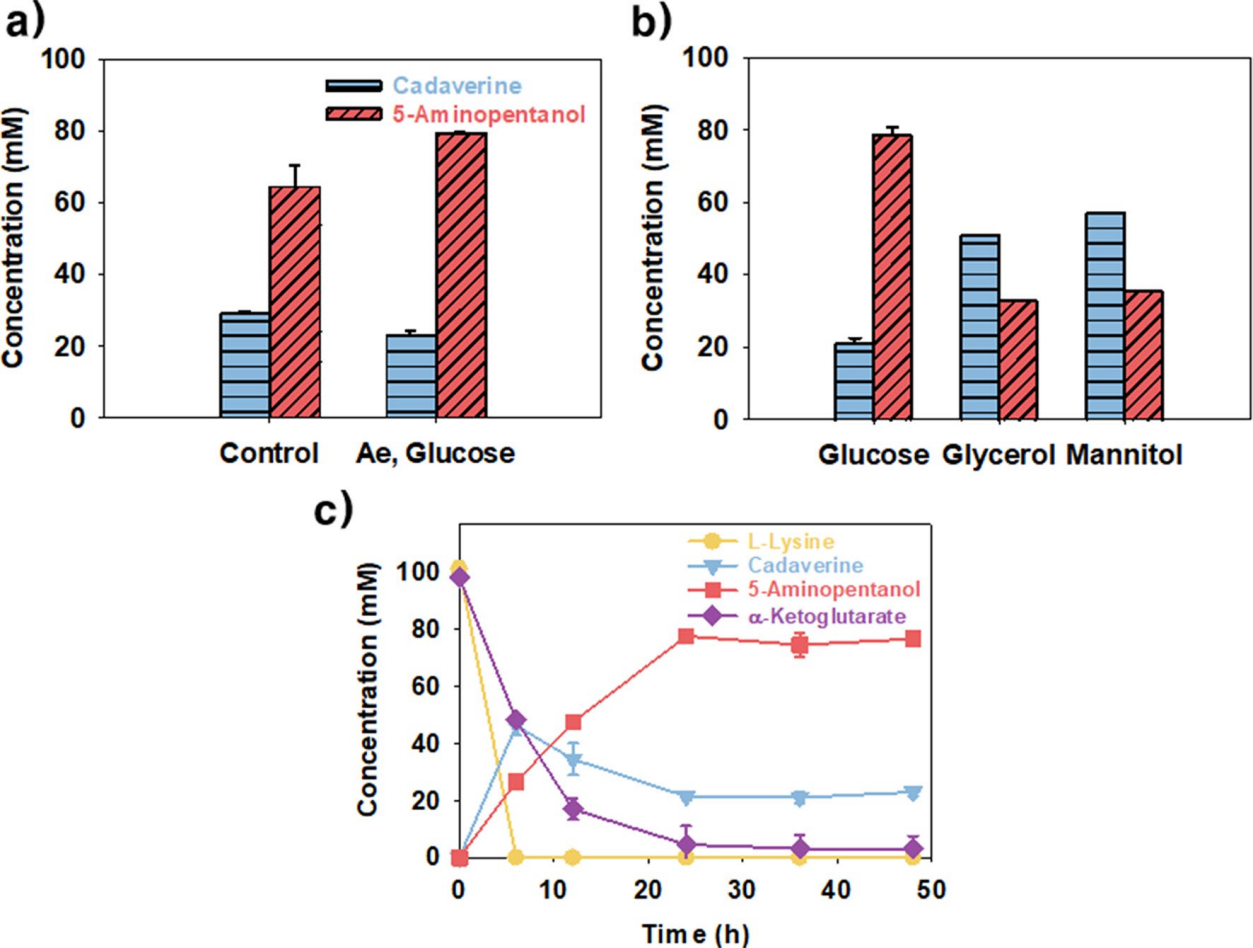
Indirect supply of α -KG through carbon source metabolism. **(a)** Effect of increased aeration and glucose supplementation on 5-AP production. Control shows the condition without glucose, Ae, glucose indicates the condition with 120 mM glucose and aeration simultaneously. **(b)** Comparison of 5-AP production using different carbon sources (glucose, glycerol, and mannitol). **(c)** Time-course profile of 5-AP production and cadaverine accumulation in response to 2% glucose supplementation

Previous studies have reported that direct supplementation of α-KG is insufficient to sustain the transamination, as α-KG is rapidly metabolized through the citric acid cycle, limiting its availability for aminotransferase activity (Wang et al. 2017; Cha et al. 2022). To address this, we evaluated the effect of indirectly supplying α-KG through glucose metabolism to enhance 5-AP production. Furthermore, to enhance citric acid cycle activity and α-KG generation, aeration was increased by replacing the cap of the Falcon tube with a perforated parafilm. When the reaction was performed using the *E. coli* AP_T7_Triple_ strain under 30 °C in Tris-HCl buffer (pH 6.5) conditions with the supplementation of 120 mM glucose, 78.5 ± 1.2 mM 5-AP was successfully synthesized, accompanied by a reduction in cadaverine accumulation to 20 ± 0.9 mM (Fig. 6a and c). Compared with the reaction without aeration and glucose supplementation, which resulted in 68.5 ± 4.2 mM 5-AP at 24 h, production increased by 1.14-fold. Based on these results, we replaced glucose with glycerol and mannitol, which are known to enhance the reducing power (Murarka et al. 2008; Ortjohann and Schönheit 2024). Contrary to our expectations, 5-AP production did not improve. Only 32.8 ± 0.2 and 35.5 ± 0.1 mM 5-AP were synthesized when using glycerol and mannitol as carbon sources, respectively. Additionally, cadaverine accumulation increased, with 51.0 ± 0.1 and 57.0 ± 0.1 mM of cadaverine accumulating when fed with glycerol and mannitol, respectively (Fig. 6b). In a previous study, 5-AP was reported as an intermediate and was produced at a low yield of 17.5%, suggesting poor selectivity (Ma et al. 2025). In contrast, this study demonstrates a highly selective 5-AP production system, achieving 79.7 ± 1.2% selectivity and a 78.5 ± 1.2% yield by systematic engineering shown in Table 1. Taken together, we established a system for the selective production of 5-AP, although the PatA-mediated transamination remains incomplete and still offers room for further improvement. Moving forward, systematic engineering strategies aimed at enhancing both the catalytic efficiency and expression level of PatA will be critical for developing a more efficient production platform, ultimately enabling industrial application with high product selectivity and concentration.

**Table 1:** Comparison of 5-aminopentanol production capability

Chemically 5-AP production						
Substrate	Catalyst	Conversion (%)		Conversion selectivity(%)		Ref
2-hydoxytetrahydropyran	Ni_2_CO_1_/Al_2_O_3_	100		79		Yang et al. 2024
2-hydoxytetrahydropyran	40Ni-LDO	100		93.1		Li et al. 2020
**Bio-based 5-AP production**						
**Strains**	**Production method**	**Substrate (mM)**	**Product (mM)**	**Conversion yield (%)**	**Co-factors (mM)**	**Ref**
ML21	Flask fermentation	Glucose (83.2)	5-AP (14.5)	17.5		Ma et al. 2025
***E. coli*** **AP**_**T7_Triple**_	Whole-cell bioconversion	l-lysine (100)	5-AP (78.5)	78.5	α-KG (100), PLP (0.1) NADPH (10) Glucose (122)	This study

## Conclusion

In this study, the first selective biological production pathway of 5-AP from l-lysine was successfully established in *E. coli*. Among the tested enzymes, YqhD was identified as the most effective reductase, and its combination with LdcC and PatA enabled the initial biosynthesis of 5-AP. The transition to a T7-based dual-plasmid expression system and increasing the copy number of *patA* gene significantly improved the production efficiency while reducing cadaverine accumulation. Additional enhancement was achieved through glucose supplementation and improved aeration, which supported cofactor regeneration via central metabolism. In contrast, alternative carbon sources such as glycerol and mannitol were less effective. Overall, this l-lysine-based biosynthetic strategy offers a scalable and environmentally friendly route for 5-AP production. A summary of 5-AP conversion yield under different reaction conditions and strains is provided in Table 1. This study is expected to contribute to the future development of microbial platforms for the sustainable and selective production of 5-AP, facilitating its applications in biopolymer synthesis as a monomer of PUE and as a precursor of δ-valerolactam for nylon-5 polymerization. These polymers have potential for usage in biomedical applications, packaging, and the fiber industry.

## Electronic supplementary material

Below is the link to the electronic supplementary material.

Supplementary Material 1

Supplementary Material 2

## Data Availability

All data generated or analyzed during this study are included in this published article.
