# Supplementary figures and images for: Recombinant Escherichia coli-driven whole-cell bioconversion for selective 5-Aminopentanol production as a novel bioplastic monomer
Source: Bioresour Bioprocess. 2025 Jun 10;12(1):58. doi: 10.1186/s40643-025-00904-6 (PMC12149034; doi:10.1186/s40643-025-00904-6)

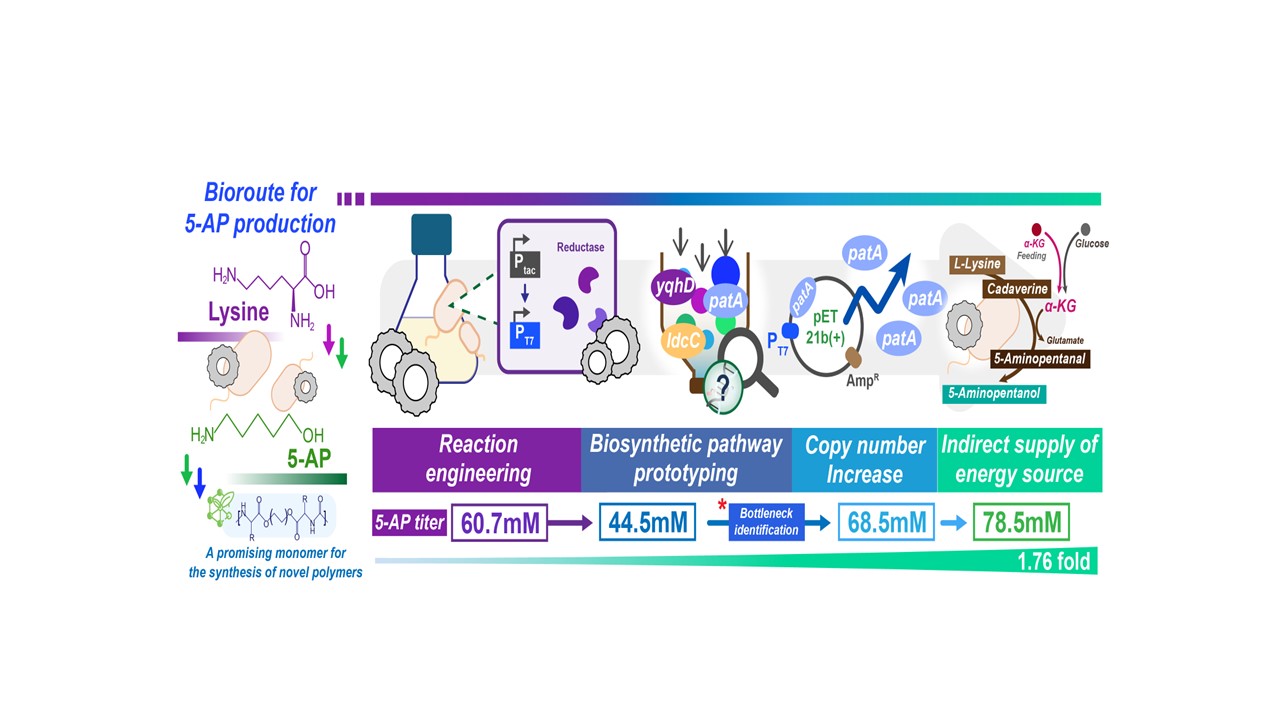

Supplement: Supplementary file 1 — Supplementary Material 1 [file 40643_2025_904_MOESM1_ESM.jpg]
