# Supplementary material for: Recombinant Escherichia coli-driven whole-cell bioconversion for selective 5-Aminopentanol production as a novel bioplastic monomer
Source: Bioresour Bioprocess. 2025 Jun 10;12(1):58. doi: 10.1186/s40643-025-00904-6 (PMC12149034; doi:10.1186/s40643-025-00904-6)
Supplement: Supplementary file 2 — Supplementary Material 2 [file 40643_2025_904_MOESM2_ESM.docx]

Supplementary Figure caption

**Supplementary Figure 1** Plasmid maps of used in this study **(a)** pKM212::*yahK*::*patA*::*ldcC*, **(b)** pKM212::*yihU*::*patA*::*ldcC*, **(c)** pKM212::*yqhD*::*patA*::*ldcC*, **(d)** pET24ma::*ldcC*, **(e)** pCDFDuet-1::*yqhD*::*patA*, **(f)** pET21b(+)::*patA*

**Supplementary Figure 1**


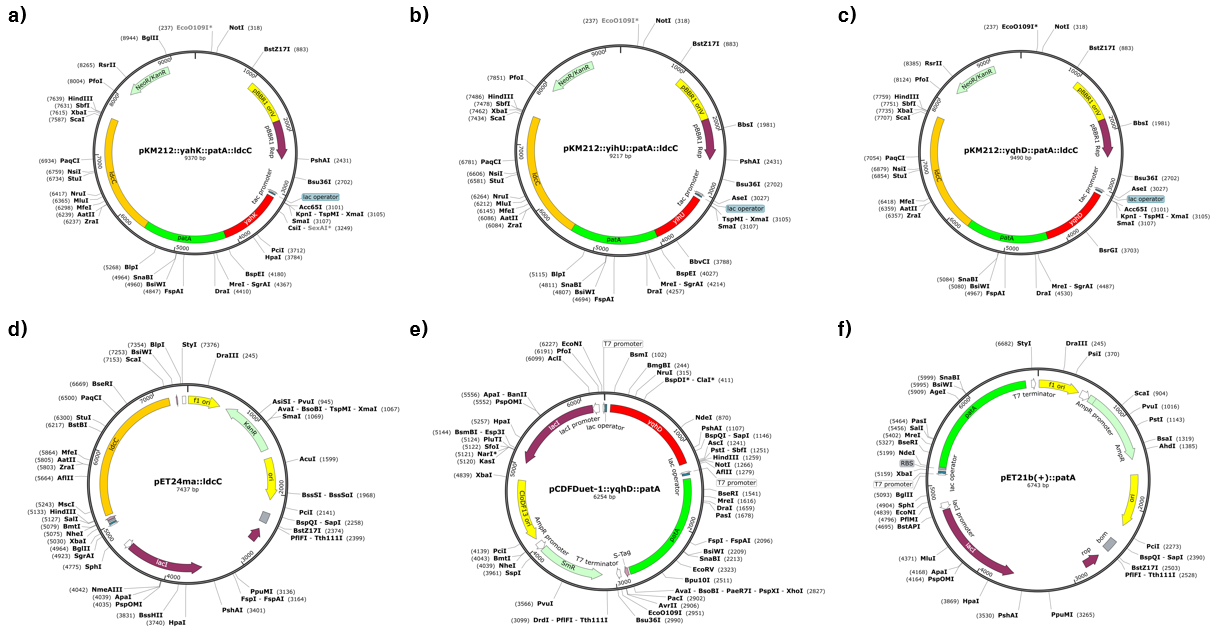


**Supplementary Table caption**

**Supplementary Table 1** Information of genes used in this study

**Supplementary Table 2** List of primers used this study

**Supplementary Table 3** List of strains and plasmids used in this study

**Supplementary Table 1**

| **Genes** | **Organism** | **Accession number** |
| --- | --- | --- |
| ***ldcC*** | ***Escherichia coli* str. K-12 substr*.* MG1655** | **NC_000913.3**  **Region: 209679…211820** |
| ***patA*** | ***Escherichia coli*** | **N/A** |
| ***yahK*** | ***Escherichia coli* str. K-12 substr*.* MG1655** | **NC_000913.3**  **Region: 342884…343933** |
| ***yihU*** | ***Escherichia coli* str. K-12 substr*.* MG1655** | **NC_000913.3**  **Region: 4072675…4073571** |
| ***yqhD*** | ***Escherichia coli* str. K-12 substr*.* MG1655** | **NC_000913.3**  **Region: 3155355…3156518** |

**Supplementary Table 2**

| **Genes & vectors** | **Primers sequence (5’ to 3’)** | **Used enzyme** | **Ref** |
| --- | --- | --- | --- |
| ***ldcC*** | **F: GGATCC *TTTCACACAGGAAACAGACC* ATGAACATCATTGCCATTATGGGACCG**  **R**: **CCTGCAGG TTATCCCGCCATTTTTAGGACTCGTACG** | **F: BamHI**  **R: SbfI** | **This study** |
| ***ldcC*** | **F: CGTCGT AAGCTT GCATGAACATCATTGCCATTATGGA**  **R**: **CGTCGT CTCGAG TCCCGCGATTTTTAGGACTCG** | **F: HindIII**  **R: XhoI** | **(Shin et al. 2018)** |
| ***patA*** | **F: GGTACC *TTTCACACAGGAAACAGACC* ATGATACGCGAGCCTCCGGAG**  **R**: **GGATCC TCACGCTTCTTCGACACTTACTCGC** | **F: KpnI**  **R: BamHI** | **This study** |
| ***patA*** | **F:** **gaaggagatatacatatgatacgcgagcctcc**  **R:** **AGCAGCCGGATCTCACGCTTCTTCGACACTTAC** | **Gibson Assembly^®^**  **Master Mix** | **This study** |
| ***yahK*** | **F: GAATTC ATGAAGATCAAAGCTGTTGGTGCATATTCC**  **R**: **GGTACC TTAGTCTGTTAGTGTGCGATTATCGATAACAAAAC** | **F: EcoRI**  **R: KpnI** | **This study** |
| ***yihU*** | **F: GAATTC ATGGCAGCAATCGCGTTTATCGG**  **R**: **GGTACC** **TTACATTTTTACTTTGGCAGTCATCCCGG** | **F: EcoRI**  **R: KpnI** | **This study** |
| ***yqhD*** | **F: GAATTC ATGAACAACTTTAATCTGCACACCCCAAC**  **R**: **GGTACC** **TTAGCGGGCGGCTTCGTATATACG** | **F: EcoRI**  **R: KpnI** | **This study** |
| ***yqhD*** | **F: actttaataaggagatatacCatgaacaactttaatctgcaca**  **R**: **acctgcaggcgcgccgagctTTAGCGGGCGGCTTCGTA** | **Gibson Assembly^®^**  **Master Mix** | **This study** |
| **pCDFDuet-1** | **F: gtataagaaggagatatacaatgatacgcgagcctccg**  **R: tttaccagactcgagggtacTCACGCTTCTTCGACACTTA** | **Gibson Assembly^®^**  **Master Mix** | **This study** |
| **pET21b(+)** | **F:** **tgagatccggctgctaac**  **R:** **ATGTATATCTCCTTCTTAAAGT** | **Gibson Assembly^®^**  **Master Mix** | **This study** |

**Supplementary Table 3**

| **Strains and plasmids** | **Genotype of strain and plasmid** | **Strategies** | **Ref or source** |
| --- | --- | --- | --- |
| **Strains** |  |  |  |
| ***E. coli* DH5α** | **F’ Φ80lacZ• ΔM15 •ƒ(lacZYAargF) U169 deoR recA1 endA1 hsdR17(rk-, mk+) phoA supE44 thi-1 gyrA96 relA1** | **Gene manipulation** | **Enzynomics** |
| ***E. coli* BL21(DE3)** | **F- dcm ompT hsdS(rB- mB-) gal λ(DE3)** | **Preparation of whole-cell bioconversion** | **Enzynomics** |
| ***E. coli* AP_YahK_** | ***E. coli* BL21(DE3) harboring pKM212::*yahK*::*patA*::*ldcC*** | **Development of 5-AP conversion system and reductase screening** | **This study** |
| ***E. coli* AP_YihU_** | ***E. coli* BL21(DE3) harboring pKM212::*yihU:*:*patA*::*ldcC*** | **Development of 5-AP conversion system and reductase screening** | **This study** |
| ***E. coli* AP_YqhD_** | ***E. coli* BL21(DE3) harboring pKM212::*yqhD*::*patA*::*ldcC*** | **Development of 5-AP conversion system and reductase screening** | **This study** |
| ***E. coli* AP_T7_Dual_** | ***E. coli* BL21(DE3) harboring pET24ma::*ldcC* and**  **pCDFDuet-1::*yqhD*::*patA*** | **Expression system change** | **This study** |
| ***E. coli* AP_T7_Triple_** | ***E. coli* BL21(DE3) harboring pET24ma::*ldcC*,**  **pCDFDuet-1::*yqhD*::*patA*, pET21b(+)::*patA*** | **Enhancing PatA mediated transamination** | **This study** |
| **Plasmids** |  |  |  |
| **pKM212::*yahK*:: *patA*::*ldcC*** | **pKM212 derivative; *yahK*, *patA,* *ldcC*, Km^R^** | **Development of 5-AP conversion system and reductase screening** | **This study** |
| **pKM212::*yihU*:: *patA*::*ldcC*** | **pKM212 derivative; *yihU*, *patA*, *ldcC*, Km^R^** | **Development of 5-AP conversion system and reductase screening** | **This study** |
| **pKM212::*yqhD*:: *patA*::*ldcC*** | **pKM212 derivative; *yqhD*, *patA*, *ldcC*, Km^R^** | **Development of 5-AP conversion system and reductase screening** | **This study** |
| **pET24ma::*ldcC*** | **pET24ma derivative; *ldcC*, Km^R^** | **Expression system change** | **(Shin et al. 2018)** |
| **pCDFDuet-1:: *yqhD*::*patA*** | **pCDFDuet-1 derivative; *yqhD* and *patA*, Sm^R^** | **Expression system change** | **This study** |
| **pET21b(+)::*patA*** | **pET21b(+) derivative; *patA*, Amp^R^** | **Enhancing PatA mediated transamination** | **This study** |
